# Deciphering treatment patterns in non-severe/moderate aplastic anemia: an international observational study

**DOI:** 10.1038/s41375-023-02047-z

**Published:** 2023-10-04

**Authors:** Bruno Fattizzo, Carmelo Gurnari, Giulio Cassanello, Marta Bortolotti, Hussein Awada, Sabrina Giammarco, Dario Consonni, Simona Sica, Shreyans Gandhi, Roochi Trikha, Joanna Large, Sarah Salter, Jaroslaw P. Maciejewski, Wilma Barcellini, Austin G. Kulasekararaj

**Affiliations:** 1 Department of Oncology and Hemato-Oncology, University of Milan, Milan, Italy; 2 Fondazione IRCCS Ca’ Granda Ospedale Maggiore Policlinico, Milan, Italy; 3 Department of Translational Hematology and Oncology Research, Taussig Cancer Institute, Cleveland Clinic, Cleveland, OH USA; 4 Department of Biomedicine and Prevention, University of Rome Tor Vergata, Rome, Italy; 5 Dipartimento di Diagnostica per Immagini, Radioterapia Oncologica ed Ematologia, Fondazione Policlinico Universitario A. Gemelli IRCCS, Roma, Italy; 6 Department of Hematological Medicine, King’s College Hospital, London, UK; 7 Faculty of Life Sciences and Medicine, King’s College London, London, UK

**Keywords:** Anaemia, Autoimmune diseases

## Abstract

Non-severe aplastic anemia is a rare bone marrow failure disorder characterized by variable degrees and combination of cytopenias, with limited data on management and outcome. We describe a large multicentric series of 259 patients, focusing on clinical and molecular features, treatment, evolution, and survival. The majority required treatment with cyclosporine (CyA) alone (*N* = 84) or in combination with anti-thymocyte globulin (ATG,44) or eltrombopag (20), eltrombopag alone (10), or others (25) including androgens. Similar outcomes were observed across different strategies, with a 6-month overall response rate of 73% for CyA, 74% for ATG plus CyA, 68% for CyA plus eltrombopag, 87% for eltrombopag, and 79% for others. Notably, 56 patients (39%), mainly receiving CyA plus eltrombopag, achieved a trilineage response (*p* = 0.02). Progression to myeloid neoplasms was limited (8%) and not related to mutational status. Hemolytic PNH developed in 10% of cases, being predicted by detection of small clones at diagnosis. Survival was negatively impacted by age, male gender, LDH, platelets/erythrocyte transfusion need, and somatic mutations by NGS, and positively by higher neutrophils at diagnosis, PNH clones, and trilineage response at 6 and 12 months. Multivariable analysis confirmed the detrimental role of age and the favorable association with PNH clone and trilineage response at 6 months.

## Introduction

Non-severe aplastic anemia (NSAA), also called “moderate” AA, is a rare bone marrow failure (BMF) disorder characterized by a variable degree and combination of cytopenias [1]. For severe cases, the treatment algorithm is more clearly established and encompasses either hematopoietic stem cell transplant or immunosuppression (IST, i.e., association of cyclosporine, CyA, and anti-thymocyte globulin, ATG) with or without the thrombopoietin receptor agonist (TPO-RA) eltrombopag, based on patients’ age, fitness, and availability of suitable donors [1–3]. For NSAA, the precise criteria establishing when to start treatment are less obvious, recommendations for the clinical workup and therapeutic strategies are largely lacking, and the final management is primarily based on individual centers’ expertise. Additionally, recent reports highlighted the heterogeneity of the disease and the phenotypic overlap with other entities characterized by cytopenias such as inherited BMF and hypoplastic myelodysplastic syndromes (MDS), which differ for treatment requirement, response to IST, and risk of evolution into aggressive myeloid neoplasms [4–6]. Besides myeloid evolution, paroxysmal nocturnal hemoglobinuria (PNH) clones may emerge as a secondary event in patients with NSAA or be present at disease onset. In such a case, PNH clones represent the immunological stigmata of the disease, constituting a cross-sectional event throughout the full spectrum of “hypocellular” bone marrow failure syndromes [2, 7]. However, the risk of evolution into hemolytic PNH and MDS as well as potential clinical predictors of outcomes are ill-defined in NSAA [5]. Here, we describe a large multicentric series of NSAA patients focusing on their clinical and molecular features, treatment needs, disease evolution, and survival.

## Patients and methods

We accrued a total of 259 patients diagnosed with NSAA at four tertiary hematologic centers in Italy (Milan and Rome), USA, and UK between 1980 and 2022. Patients diagnosed with inherited bone marrow failures by clinical suspicion (i.e., family history, young age at presentation, clinical features, etc.) coupled with telomere length (mostly done by qPCR) and inherited BMF screen (including chromosome fragility test) were excluded from the analysis. NSAA was classified according to modified Camitta criteria as AA not fulfilling the criteria for severe or very severe (VS)AA (i.e. marrow cellularity <25%, plus at least 2 of (i) neutrophils <0.5 × 10^9/L, (ii) platelets <20 × 10^9/L (iii), reticulocyte count <60 × 10^9/L) [1]. A minimum follow-up of 6 months was required to be included in the study.

The study was conducted according to the Declaration of Helsinki and approved by the local Ethical Committee of each participating center.

Details on baseline hematologic features, bone marrow trephine, flow-cytometry (FC) of marrow aspirates and mutational status by next-generation sequencing (NGS) of common myeloid drivers were reviewed where available (see also Supplementary Table 1). In the flow-cytometry analysis, indicators of a T-cell or B-cell clonal lymphoproliferative process included absent, diminished, or abnormally increased expression of cell antigens, along with subset restriction, and the presence of aberrant antigens. In case of one of the previous findings was present, confirmation of clonality was performed by PCR or flow cytometry of TCR repertoire for T-cells, whilst for B-cells Ig restriction was used. FC data were available in a proportion of patients only mainly due to test not performed or *punctio sicca*.

The different management strategies were registered and categorized as: 1) cyclosporin plus/minus steroids (CyA), 2) ATG plus CyA, 3) eltrombopag plus CyA, 4) eltrombopag single agent, and 4) other. Notably, in thrombocytopenic patients, therapy was generally started when PLT counts were <50 × 10^9/L, whilst isolated neutropenia per se was not used as intention to treatment.

Responses were assessed at 6 and 12 months according to European Bone Marrow Transplant group criteria (complete, CR, if platelets PLT > 100 × 10^9/L, hemoglobin Hb >10 g/dL, neutrophils-ANC > 1.5 × 10^9/L; partial, PR, if transfusion independence but not meeting the above-defined criteria) [1, 3].

All relapses were registered, as well as adverse events, including infectious, thrombotic, and bleeding complications, graded according to the Common terminology criteria CTCAE version 5. The occurrence of evolution into hemolytic PNH, severe AA, and myeloid neoplasms (namely MDS or acute myeloid leukemia - AML), as well as death and the relative causes were also recorded for all patients.

### Statistical analysis

Kruskal-Wallis and chi-squared tests were used for comparison of quantitative and categorical variables, respectively. We performed survival analysis after truncating follow-up time (time since diagnosis to last follow-up) at 15 years. We analyzed overall survival (OS) and mortality using the Kaplan-Meier estimator. Hazard ratios (HR) and 95% confidence intervals (CI) were calculated for selected variables using univariate and multivariable Cox regression models (time axis: time since diagnosis). When evaluating clinical response at 6 or 12 months, time axis started 6 or 12 months after diagnosis, respectively. The multivariable model contained the covariate age (continuous, in decades), PNH clone, and trilineage response at 6 months. We graphed cumulative incidence curves for myeloid neoplasms and PNH; death and each of these two conditions were treated as reciprocal competing events. For the analysis of predictors of outcome, the statistical significance in univariate and multivariate analysis was calculated only for patients with complete available data. Statistical analyses were performed with Stata 17 (StataCorp. 2021).

## Results

### Demographic and hematologic features at diagnosis

As shown in Table 1, median age of our cohort was 50 years (range, 6–91 years, IQR 35). At baseline, 88% of cases presented with thrombocytopenia (PLT < 100  ×  10^9/L; 70% < 50  ×  10^9/L, 22% <20 × 10^9/L), 57% with anemia (Hb <10 g/dL; 16% <8 g/dL), and 34% with neutropenia (ANC < 1 × 10^9/L, 8% <0.5 × 10^9/L). Sixty-three patients had bicytopenia (47 PLT and Hb, 15 PLT and ANC, and 1 Hb and ANC), and 41 pancytopenia. Regarding transfusions, 127 patients were transfusion dependent (81, 37% for PLT, 116, 53% for red blood cells (RBC), and 70, 27% for both). Bone marrow evaluation showed a median cellularity of 15% (5–60), being hypocellular for age in all subjects, with grade 1–2 reticulin fibrosis (MF-1/2) in 55/159 (35%) evaluated patients. FC (*N* = 160) revealed a polyclonal T-cell infiltrate in 35 patients (22%), mixed T- and B-cell in 25 (16%), B-cell in 3 (2%), and no infiltrate in the others. PNH clone was detected by FC in 109 patients (49%, 222 tested), with a median granulocyte clone size of 1.5% (0.1–99). Cytogenetics was abnormal in only 14 patients, and not evaluable (no growth) in 3 cases. None of the patients reached the WHO 2016 or 2022 criteria for MDS (i.e., bone marrow dysplasia >10%, MDS-defining cytogenetics or molecular aberration) [8, 9]. Baseline NGS (*N* = 132) showed at least one mutation in 22 patients (4 in *BCOR*, 5 in *TET2*, 4 in *ASXL1*, 3 in *DNMT3A*, and 1 each in *CUX1*, *SRSF2*, *U2AF1, NRAS, CBL, EZH2, CALR, ETV6, RAD21, EP300, MPL, SBDS, NF1*), 2 mutations in 5, and 3 mutations in 2 cases (see also Supplementary Table 1 for a full report of mutational details).

**Table 1:** Baseline features of non-severe aplastic anemia (NSAA) patients. Values are given as median (range) unless otherwise specified.

Baseline features	Untreated	CyA	ATG + CyA	CyA + EPAG	EPAG	Other	All patients
*N*	76	84	44	20	10	25	259
Male/Female	28/48	36/48	22/22	11/9	5/5	12/13	114/145
Age, years*	36 (7–85)	57 (14–91)	43 (16–71)	68 (21–83)	70 (29–80)	36 (6–86)	50 (6–91)
Hb, g/dL	11.7 (7.7–15.1)	9.8 (4.5–14.7)	9.4 (7–13.8)	8.5 (5.2–14.3)	8.6 (6.3–11.9)	9.2 (5.6–11.8)	9.5 (4.5–15.1)
PLT ×10^9/L**	86 (31–216)	31 (4–182)	26 (7–102)	24 (5–53)	24 (3–43)	30 (8–210)	34 (4–216)
ANC ×10^9 L	1.6 (0.4–7.2)	1.16 (0.03–8.8)	1.09 (0.6–2.7)	1.3 (0.5–4.9)	1.21 (0.26–2.5)	1.15 (0.37–6.54)	1 (0.03–8.8)
Ret ×10^9/L	60 (21–134)	59 (4–57)	60 (20–80)	50 (20–110)	50 (10–140)	40 (20–63)	52 (4–140)
LDH U/L	206 (135–1947)	221 (94–1036)	218 (159–300)	220 (140–347)	192 (106–799)	211 (148–357)	240 (94–1947)
endogenous EPO U/L	88 (12–1438)	524 (3.9–5032)	534 (69–1865)	151 (85–1248)	240 (56–728)	383 (94–1053)	380 (3.9–5032)
PNH clone, *N*	22/59	36/77	26/35	9/20	5/10	11/21	109/222
Size, %	7.2 (0.05–99.4)	2.5 (0.0–93.3)	1.4 (0–13)	0.9 (0.01–16.3)	0 (0–2)	4.5 (0–99)	1.5 (0.1–99)
RBC transfusion, N**	5/39	59/84	15/40	12/20	10/10	15/25	116/218
PLT transfusion, N**	1/39	42/84	10/40	9/20	8/10	11/25	81/218
Trephine cellularity, %	15 (2–40)	10 (3–50)	10 (1–30)	10 (3–30)	20 (3–40)	10 (3–60)	15 (5–60)
MF-0, *N*	21/31	48/71	10/19	13/16	5/9	7/13	104/159
MF-1, *N*	10/31	21/71	8/19	3/16	4/9	6/13	52/159
MF-2, *N*	0/31	2/71	1/19	0/16	0/9	0/13	3/159
Lymphoid infiltrate, *N*	11/31	25/71	5/19	9/16	7/9	6/14	63/160
T/B/mixed, *N*	10/0/1	9/1/15	4/1/0	7/0/2	2/1/4	3/0/3	35/3/25
Abnormal karyotype, *N*	3/66	2/83	5/40	2/19	0/10	2/23	14/241
Abnormal NGS, *N*	6/36	5/47	0/8	4/18	2/7	5/16	22/132

Endogenous erythropoietin was not available in 12 cases, karyotype in 18, FC in 99, and NGS in 127 patients. For 41 patients, mainly untreated and mildly cytopenic, transfusion records were not available from clinical charts.

*CyA* cyclosporine, *ATG* anti-thymocyte globulin, *EPAG* eltrombopag, *Hb* hemoglobin, *PLT* platelets, *ANC* absolute neutrophil counts, *Ret* reticulocytes, *LDH* lactate dehydrogenase, *EPO* erythropoietin, *PNH* paroxysmal nocturnal hemoglobinuria, *RBC* red blood cell, *NGS* next-generation sequencing.

*p < 0.01; **p < 0.001.

### Management and first-line treatment strategies

Over a median follow-up time of 48 months (6–278), 76 patients (29%) were judged eligible to observation only. These patients were significantly younger (*p* < 0.01), transfusion-free (*p* < 0.001) and had higher PLT counts (*p* < 0.001; Table 1) compared to those requiring treatment. Reasons for treatment initiation were transfusion dependence (*N* = 117), moderate-to-severe anemia and thrombocytopenia (*N* = 48) or neutropenia (*N* = 12), and not reported (*N* = 6). Treatments included CyA (46%), CyA plus ATG (24%), CyA plus eltrombopag (11%), eltrombopag alone (5%), and other treatments (14%; *N* = 16 androgens, 3 alemtuzumab, 2 daclizumab, 1 azathioprine, and 3 tacrolimus). The different treatments were not chosen depending on age and were equally distributed across all age decades.

The overall response rate (CR + PR) at 6 months in evaluable patients (*N* = 144) was 73% for CyA, 74% for ATG plus CyA, 68% for CyA plus eltrombopag, 87% for eltrombopag, and 79% for others, without significant differences across treatment groups. The distribution of PLT, Hb, and ANC responses is detailed in Table 2 (*N* = 133 patients with detailed data), with 24 patients (18%) achieving a bilineage and 55 (41%) a trilineage response. Notably, patients receiving CyA plus eltrombopag had a higher rate of trilineage response (63 versus 37% with other treatment modalities, *p* = 0.02).

**Table 2:** Hematologic response according to different treatment strategies.

	Untreated *N* = 76	CyA *N* = 84	ATG + CyA *N* = 44	CyA + EPAG *N* = 20	EPAG *N* = 10	Other *N* = 25	All treated *N* = 183
Time to treatment, months	/	2 (0–102)	2 (0–30)	2 (0–2)	0 (0–8)	1 (0–120)	2 (0–120)
Median follow up, months	87 (6–218)	74 (8–210)	112 (11–278)	25 (6–99)	41 (6–56)	43 (12–113)	46 (6–278)
*Response at 6 months (N* = *144)*							
Overall response, *N*(%)	/	55/75 (73)	20/27 (74)	13/19 (68)	7/8 (87)	12/15 (79)	108/144 (75)
PLT response, *N*	/	74	19	19	8	12	132
CR *N* (%)	/	17 (23)	4 (21)	6 (32)	0	3 (25)	30 (23)
PR *N* (%)	/	25 (34)	10 (53)	7 (36)	5 (62)	6 (50)	52 (39)
Hb response, *N*	/	75	19	19	8	12	133
CR N(%)	/	30 (40)	10 (53)	9 (47)	3 (37)	7 (58)	59 (44)
PR N(%)	/	12 (16)	2 (10)	3 (16)	2 (25)	1 (8)	19 (14)
ANC response, *N*	/	75	19	19	8	12	133
CR *N* (%)	/	26 (35)	9 (47)	12 (63)	4 (50)	3 (25)	54 (41)
PR *N* (%)	/	12 (16)	5 (26)	0	1 (12)	4 (33)	22 (17)
Extent, *N*	/	75	19	19	8	12	133
Monolineage *N*(%)	/	12 (16)	0	1 (5)	2 (25)	1 (8)	17 (13)
Bilineage *N*(%)	/	18 (24)	1 (5)	0	2 (25)	3 (25)	24 (18)
Trilineage *N*(%)	/	26 (35)	8 (42)	12 (63)*	3 (37)	6 (50)	55 (41)
*Response at 12 months (N* = *119)*							
Overall response, N(%)	/	56/63 (89)	23/31 (74)	14/15 (93)	3/3 (100)	6/7 (86)	102/119 (86)
PLT response, *N*	/	57	18	15	3	6	99
CR *N*(%)	/	23 (40)	7 (39)	7 (47)	2 (67)	2 (33)	41 (41)
PR *N*(%)	/	28 (49)	10 (55)	7(47)	1 (33)	3 (50)	49 (49)
Hb response, *N*	/	57	18	15	3	6	99
CR *N*(%)	/	39 (68)	13 (72)	11 (73)	2 (67)	5 (83)	70 (70)
PR *N*(%)	/	11 (19)	1 (5)	2 (13)	1 (33)	1 (16)	16 (16)
ANC response, *N*	/	57	18	15	3	6	99
CR *N*(%)	/	35 (61)	10 (55)	12 (80)	3 (100)	2 (33)	62 (62)
PR *N*(%)	/	12 (21)	6 (33)	2 (13)	0	2 (33)	22 (22)
Extent, *N*	/	57	18	15	3	6	99
Monolineage *N*(%)	/	1 (2)	1 (5)	0	0	1 (16)	3 (3)
Bilineage *N*(%)	/	9 (16)	2 (11)	1 (7)	0	0	12 (12)
Trilineage *N*(%)	/	44 (77)	14 (78)	13 (87)	3 (100)	5 (83)	79 (79)
Adverse events, *N*	/	24/84	10/44	4/20	0/10	0/25	38/183
Therapy stop, *N*	/	45/84	22/44	7/20	1/10	4/25	79/183
Response *N*(%)	/	10 (22)	11 (50)	3 (43)	1 (100)	2 (50)	27 (34)
NR *N*(%)	/	12 (27)	3 (14)	3 (43)	/	2 (50)	20 (25)
AEs *N*(%)	/	17 (38)	8 (36)	1 (14)	/	/	26 (33)
Death *N*(%)	/	5 (11)	0	0	/	/	5 (6)
NA *N*(%)	/	1 (2)	/	/	/	/	1 (2)
Evolution, *N*(%)	13/76 (17)	15/84 (18)	11/44 (25)	5/20 (5)	2/10 (20)	2/25 (8)	48/259 (18)
AA, *N*	0	1	0	1	1	1	4
AML, *N*	2	1	1	0	0	0	4
MDS, *N*	4	4	6	1	1	0	16
PNH, *N*	7	9	4	3	0	1	24
Death, *N*	9/66	19/77	5/38	2/18	3/9	4/23	42/231

Of 259 patients, 183 were treated. Of those requiring treatment, 144 and 119 had available data at 6 and 12 months for response evaluation. Single lineage responses are reported only for those patients exhibiting a specific cytopenia.

*CyA* cyclosporine, *ATG* anti-thymocyte globulin, *EPAG* eltrombopag, *Hb* hemoglobin, *PLT* platelets, *ANC* absolute neutrophil counts, *CR* complete response, *PR* partial response, *NR* no response, *NA* not available, *AA* aplastic anemia, *AML* acute myeloid leukemia, *MDS* myelodysplastic syndrome, *PNH* paroxysmal nocturnal hemoglobinuria.*p = 0.02 versus other treatment modalities.

The overall response rate (CR + PR) at 12 months in evaluable patients (*N* = 119) was 89% for CyA, 74% for ATG plus CyA, 93% for CyA plus eltrombopag, 100% for eltrombopag, and 86% for others, again without differences across groups (Table 2). Regarding single lineage response (Table 2, *N* = 99 patients with detailed data), 12 patients (12%) achieved a bilineage and 79 (79%) a trilineage response.

The calculated median duration of cyclosporin therapy was 27 months (range 6 to 54) in patients with available treatment duration (*N* = 112) including tapering after initial response and maintenance at the lowest effective dose (in some patients 25 mg twice per week).

In total, 21% of cases experienced ≥1 adverse event (AE) of grade ≥2. Grade 3/4 toxicities (*N* = 6/38) were more common in the CyA plus eltrombopag group and encompassed retinal thrombosis, gastroenteritis, diarrhea and transaminitis. Only the patient with grade 4 transaminitis required permanent discontinuation after 6 months of CyA plus eltrombopag, and treatment-free response was maintained at 2.3 years follow-up. Overall, therapy discontinuation occurred in 43% of treated subjects due to persistent CR (*N* = 29), non-response (*N* = 18), AE (*N* = 26), and death (*N* = 5), with no difference across treatment groups.

By restricting the analysis to patients diagnosed in the last 10 years (i.e., from January 2012), response rates at 6 and 12 months were comparable, with no differences regarding treatment strategies (CyA, CyA plus eltrombopag, eltrombopag alone, only eight patients received ATG after 2012). No predictors of outcome were identified.

### Further therapy lines

A total of 39 patients received multiple lines of therapy due to non-response (*N* = 32) and/or toxicity (*N* = 7) of previous treatment. Further therapies included the following agents alone or in combination (Supplementary Table 2): ATG (*N* = 14 as second line, 1 third line), eltrombopag (*N* = 9 as second line, 5 third line), androgens (*N* = 8 as second line, 2 third line), tacrolimus (*N* = 5 as second line, 2 third line), alemtuzumab (*N* = 2 as third line), hematopoietic stem cell transplant (*N* = 5 as second line, 5 third line). Of note is that 24 patients (9.2%) received anti-complement therapy for hemolytic PNH. Finally, the 76 untreated patients never required therapy for their NSAA during the study period.

### Disease evolution

**Fig. 1:**
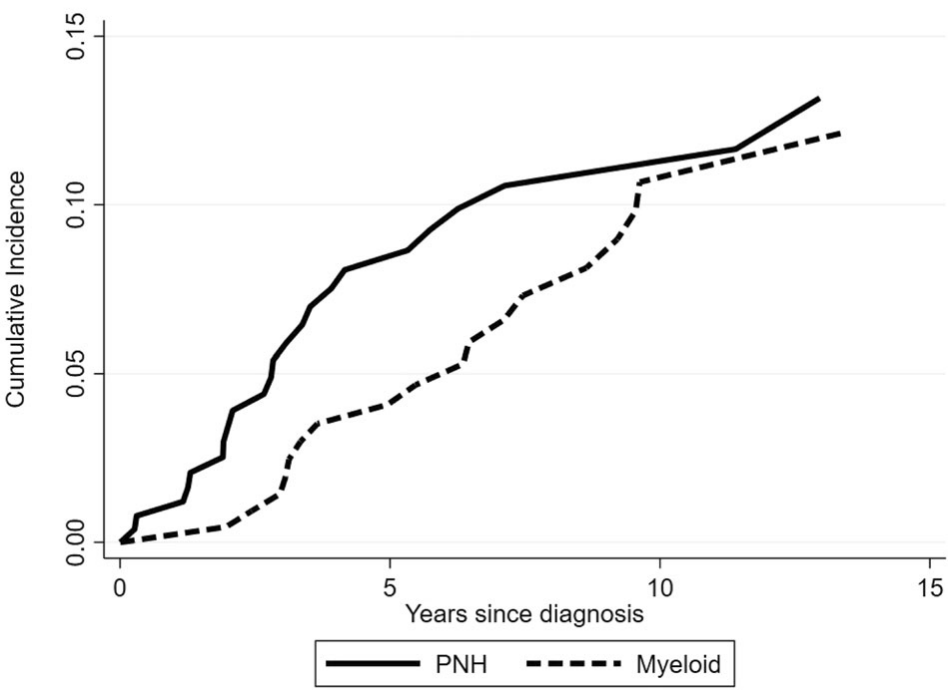
Clonal evolution in patients with non-severe aplastic anemia. Cumulative incidence of evolution of non-severe aplastic anemia into myeloid neoplasm (dashed line) and of development of hemolytic paroxysmal nocturnal hemoglobinuria (PNH, continuous line).

Over the follow-up time, 20 patients (8%) evolved to myeloid neoplasm (Cumulative incidence 12.2%; Fig. 1). Progression to MDS was registered in 16 cases (including four untreated subjects) at a median time of 6.5 years (0.6–13.6), whereas 4 evolved to AML (2 untreated subjects) at a median time of 3.2 years (2.5–5.5). The rate of clonal evolution to MDS/AML was not associated with clinical or hematologic parameters at diagnosis, nor with eltrombopag treatment.

During observation, 24 patients developed hemolytic PNH requiring anti-complement therapy. In such cases, PNH clone was either not present at diagnosis (*N* = 11) or increased to >10% of their PNH clone size at a median time of 2.7 years (0.6–23) from initial NSAA onset (Cumulative incidence of 13.2% at 15 years, Fig. 1). This was associated with the detection of small or very small clones at NSAA onset (*p* < 0.001). Finally, only four patients (all treated) had no response and eventually met the criteria for severe aplastic anemia after a median time of 7.4 years (0.8–18) from initial diagnosis.

### Survival analysis

**Fig. 2:**
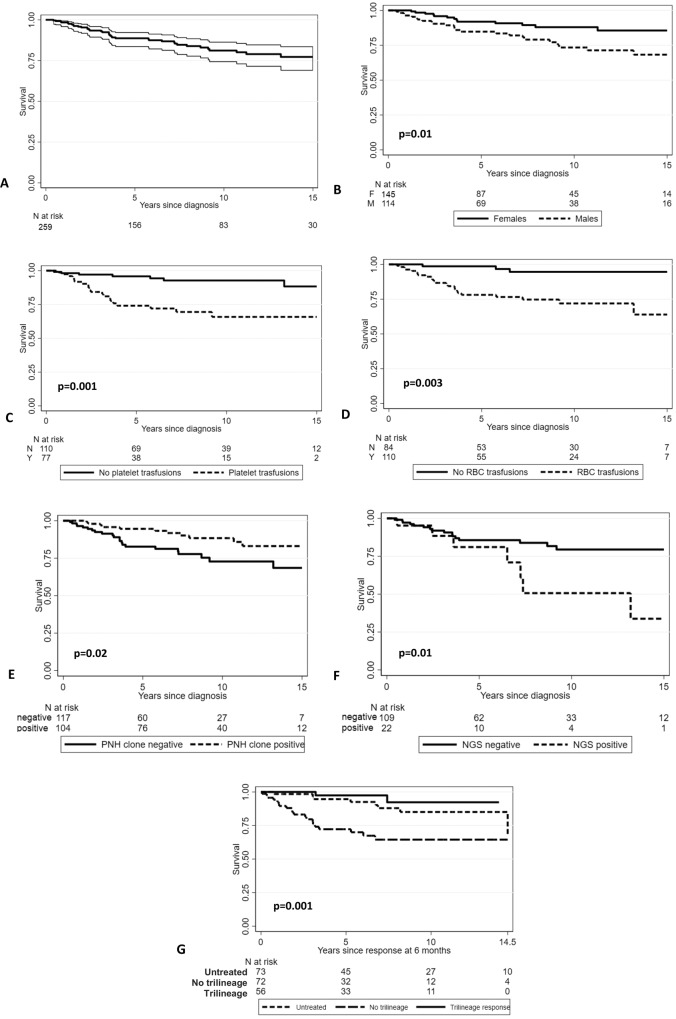
Overall survival in patients with non-severe aplastic anemia. Overall survival in patients with non-severe aplastic anemia altogether (**A**) or divided by gender (**B**), need of transfusions (**C**, **D**), presence of paroxysmal nocturnal hemoglobinuria (PNH) clone (**E**), presence of mutations by next generation sequencing (NGS, **F**), and response to treatment (**G**). RBC red blood cell.

Overall, 42 patients died during the follow-up (28 lost to follow-up, 10.8%), with infections as the leading cause of mortality (*N* = 28, 67%). The remaining patients died due to congestive heart failure/heart attack (*N* = 5), respiratory insufficiency (*N* = 3), solid cancer progression (*N* = 3), intracranial bleeding (*N* = 1), AML progression (*N* = 1), and acute GVHD (*N* = 1). Poorer survival was associated with older age [HR 1.33 (95% CI 1.1–1.5), *p* < 0.001 per 10-year increase], male gender [HR 2 (1.15–3.6), *p* = 0.01], increased LDH [HR 2.03 (1.1–3.7), *p* = 0.01], PLT and RBC transfusion dependence [3.75 (1.7–8), *p* = 0.001; and 4.7 (1.7–13), *p* = 0.003, respectively], and somatic mutations by NGS [2.33 (1.1–4.7), *p* = 0.01] at baseline (Fig. 2). Protective factors at baseline were instead higher ANC [HR 0.6 (0.4–0.9), *p* = 0.02] and presence of a PNH clone [HR 0.44 (0.2–0.9), *p* = 0.02]. Furthermore, overall response at 6 and 12 months [HR 0.25, 95%CI 0.11–0.57, *p* = 0.001; and 0.14, 0.04–0.44, *p* = 0.001, respectively] associated with better survival outcomes; more specifically, attainment of PLT [HR 0.10 (0.03–0.31), *p* < 0.001)] and Hb response at 6 months [HR 0.17 (0.06-0.46), *p* < 0.001], as well as trilineage response at 6 [HR 0.24 (0.10–0.55), *p* = 0.001] and 12 months [HR 0.17 (0.04–0.81), *p* = 0.03] were protective factors.

Beyond the detrimental effect of age [HR 1.67 (1.21–2.30), *p* = 0.002, per 10-year increase], multivariable analysis confirmed PNH clone positivity at diagnosis [HR 0.32 (0.12–0.86), *p* = 0.02], and achievement of trilineage response (complete plus partial) at 6 months (HR 0.11 (0.04–0.27), *p* < 0.001] as predictors of survival. Finally, by restricting the analysis to patients diagnosed in the last 10 years the same predictors of outcomes emerged (Supplementary Table 3).

## Discussion

This is one of the largest series ever reported highlighting treatment strategies, outcomes, and clonal evolution pattern of patients with NSAA. We demonstrate that the use of CyA alone or combined with ATG or eltrombopag leads to a significant hematologic improvement (about 70% of cases) with a favorable impact on survival. The overall response rates were similar across treatment strategies, although a higher proportion of patients treated with the CyA plus eltrombopag combination achieved a trilineage response at 6 months. Although a clear association was not observed among survival and different treatment strategies, trilineage response was found as an independent predictor for longer survival in multivariate analysis. Additionally, the number of patients receiving ATG was markedly reduced in the last decade (80% treated before 2012), making it a less preferred strategy in NSAA in the TPO-RA era. The similar outcomes obtained with CyA with or without eltrombopag suggest limiting ATG, a highly immunosuppressive strategy also requiring hospital admission, to those patients evolving to SAA. Our data well compare with those of a recent phase 2 prospective study of eltrombopag in NSAA, where about 50% of patients responded, although the study included both naïve and previously treated patients as well as inherited bone marrow failure syndromes and used less restrictive response criteria [4]. Regarding the duration of cyclosporin therapy, our data showed a median treatment of just over 2 years, including maintenance at the lowest effective dose, almost concordant with what has been suggested for severe AA.

In the present study, up to 1/3 of patients, younger and with milder cytopenias and predominantly transfusion independent, were managed with monitoring strategy without intervention, with 8% evolving to MDS or AML and 9% developing hemolytic PNH after more than 3 years from initial diagnosis. This emphasizes the need for a life-long hematologic follow-up in these subjects, with systematic PNH clone testing, and regular bone marrow re-evaluation, particularly in cases with worsening cytopenia(s). On the other hand, one could speculate that specific subgroups of patients (e.g., those younger and untreated because of a milder hematologic phenotype) might reflect a different disease ontogenesis (e.g., germline predisposition traits or autoimmunity).

For treated patients, the rate of evolution to MDS or AML, and PNH appearance was similar to untreated cases (8% and 9%, respectively), without association with eltrombopag use, as also reported in SAA [3]. Systematic bone marrow re-evaluations are advisable in this setting (i.e., at 6, 12, and 24 months) to assess cellularity and toxicity, as also suggested for SAA [1, 3].

In the era of genomics, the use of targeted sequencing is expected to inform prognostication in terms of evolution and survival. In our series, 17% of cases harbored at least one mutation in myeloid genes, similarly to a recent report [5], being associated with shorter survival in univariate analysis. However, the molecular landscape did not differ among cytopenia groups, treated or untreated patients, and did not associate with evolution to MDS or AML. In a recent experience of 85 NSAA patients, the frequency of mutations, dynamically evaluated during disease course, was significantly lower than in SAA (18 versus 56%) and the authors speculated that the proportion of genomic hits might correspond to the presence of “molecularly unstable” hematopoietic clones [5]. We may further hypothesize a difference between NSAA and SAA regarding the aggressiveness of the immune attack, as well as the bottleneck effect following IST. Consistently, IST use in NSAA patients may be limited since stem cells are still preserved and may be alternatively boosted with eltrombopag [4]. Whether the immune pathogenesis of NSAA and SAA are different, or part of a same spectrum is still a matter of debate and requires further investigation. Our study also excluded patients with constitutional BMF and hypoMDS, but a systematic study to evaluate the contribution of these entities to patients presenting with moderate cytopenias is likely to be helpful.

Regarding toxicities, about 20% of treated patients experienced AEs, mainly of low grade. Eltrombopag in combination with CyA was associated with a greater percentage of grade 3/4 AEs, although only 1 out of 4 cases required permanent discontinuation. For instance, a grade 4 transaminitis was registered in a young lady on partial response after 6 months of CyA plus eltrombopag. Despite drug withdrawal, a treatment-free response was maintained after 2 years follow up. These findings, although in a limited number of patients, warrant close monitoring of renal and liver function, already routinely suggested for SAA. Additionally, assessment of thrombotic risk pre-eltrombopag is advisable, particularly in patients with PNH clones, since thromboses have been described even in thrombocytopenic patients in this setting [10]. The use of complement inhibitor, already described in combination with AA therapy [11], may be useful to limit the risk under such circumstances, and may be started before adding eltrombopag in those with large PNH clones associated with hemolysis.

Finally, older age and transfusion dependence confirmed their detrimental effect on survival, similarly to what reported for SAA where prognosis remains age-related despite the use of TPO-RA [2, 3]. However, in our series, the achievement of trilineage response to specific treatment retained an independent favorable impact, shedding light on a potential disease-modifying effect.

Our study carries the limitations inherent to its retrospective nature, particularly the evaluation over a large time period, and the heterogeneity of treatment strategies across different eras, dependent on availability and approval of medications. This, along with the differences in laboratory tests availability and reporting across decades, accounts for missing data in some patients. For instance, NGS testing for myeloid neoplasms and bone marrow failure became widely available only recently and was therefore evaluable in half of patients only. Finally, some mutations with a VAF close to 50% (e.g., heterozygous status) might be of germline origin, although confirmation with germline tissue was not possible. This notwithstanding, it provides detailed real-life data on a large series of patients with a very rare condition, and information on long-term follow up. Furthermore, the re-analysis restricted to the last 10 years showed similar results.

In summary, we report that up to a third of patients may be monitored with follow-up only (watch and wait W&W), while the majority required treatment with IST with or without eltrombopag. Therapeutic strategies had similar outcomes, with responses in about 70% of cases, but fairly higher trilineage improvement with the TPO-RA combination, that is associated with better survival in multivariate analysis. Risk of progression to myeloid neoplasms was limited (<5%) and not related to mutational status, whilst hemolytic PNH developed in nearly 10% of cases and was predicted by the detection of small clones at diagnosis.

In conclusion, NSAA requires treatment in about two-thirds of patients, possibly rescued with cyclosporine with or without eltrombopag in ~70% of cases, whilst the use of ATG should be limited.

### Supplementary information

Supplementary tables

## Data Availability

All relevant data have been added to the article file or supplementary materials. Further information may be obtained upon reasonable request to the corresponding author.
