# Supplementary material for: Deciphering treatment patterns in non-severe/moderate aplastic anemia: an international observational study
Source: Leukemia. 2023 Oct 4;37(12):2479–85. doi: 10.1038/s41375-023-02047-z (PMC10681892; doi:10.1038/s41375-023-02047-z)
Supplement: Supplementary file 1 — Supplementary tables [file 41375_2023_2047_MOESM1_ESM.docx]

**Supplementary table 1. List of mutations identified**

| **Patient** | **N° of mut** | **Gene** | **Aa change** | **VAF** | **Gene** | **Aa change** | **VAF** | **Gene** | **Aa change** | **VAF** |
| --- | --- | --- | --- | --- | --- | --- | --- | --- | --- | --- |
| 1 | 3 | *CUX1* | p.E727X | 46% | *SRSF2* | p.P95T | 67% | *TET2* | p.H1380Y | 65% |
| 2 | 1 | *TET2* | p.E1250X | 64% |  |  |  |  |  |  |
| 3 | 1 | *CALR* | p.E381fs | 26% |  |  |  |  |  |  |
| 4 | 1 | *BCOR* | p.R1455fs | 22% |  |  |  |  |  |  |
| 5 | 1 | *BCOR* | p.M1293fs | 19% |  |  |  |  |  |  |
| 6 | 2 | *TET2* | p.P669fs | 36% | *TET2* | p.1553_1554del | 45% |  |  |  |
| 7 | 1 | *MPL* | p.P106L | 50% |  |  |  |  |  |  |
| 8 | 1 | *TET2* | p.H1380Y | 13% |  |  |  |  |  |  |
| 9 | 1 | *EZH2* | p.E197K | 44% |  |  |  |  |  |  |
| 10 | 1 | *DNMT3A* | p.R882C | 30% |  |  |  |  |  |  |
| 11 | 3 | *BCOR* | p.T1469A | 26% | *ETV6* | p.E361K | 26% | *RAD21* | p.K371R | 54% |
| 12 | 1 | *EP300* | p.R2308C | 55% |  |  |  |  |  |  |
| 13 | 1 | *NRAS* | p.G12C | 11% |  |  |  |  |  |  |
| 14 | 2 | *ASXL1* | p.G645fs | 4% | *CBL* | p.D460del | 2% |  |  |  |
| 15 | 2 | *NF1* | p.A1767fs | 4% | *U2AF1* | p.Q84P | 4% |  |  |  |
| 16 | 2 | *TET2* | p.H1380Y | 6% | *SBDS* | c.2582T>C | 48% |  |  |  |
| 17 | 1 | *DNMT3A* | p.R882C | 14% |  |  |  |  |  |  |
| 18 | 2 | *DNMT3A* | p.Y874X | 7% | *DNMT3A* | p.P904L | 14% |  |  |  |
| 19 | 1 | *BCOR* | p.L1646fs | 10% |  |  |  |  |  |  |
| 20 | 1 | *ASXL1* | p.P701fs | 9% |  |  |  |  |  |  |
| 21 | 1 | *ASXL1* | p.R965X | 26% |  |  |  |  |  |  |
| 22 | 1 | *ASXL1* | p.G646fs | 15% |  |  |  |  |  |  |

Nomenclature according to Human Genome Variation Society. VAF: variant allelic frequency; aa: amino acid; mut: mutations

List of mutations studied:

USA - *ASXL1, BCOR, BCORL, CARL, CBL, CEBPA, CUX1, DDX41, DNMT3A, EP300, ETV6, EZH2, FLT3, GATA2, IDH1, IDH2, JAK2, KDM6A, KIT, KRAS, LUC7L2, MPL, NF1, NPM1, NRAS, PHF6, PRPF8, PTPN11, RAD21, RUNX1, SETBP1, SF3B1, SMC3, SRSF2, STAG2, TET2, TP53, U2AF1, U2AF2, WT1, ZRSR2*.

Italy - *ABL1, BRAF, CBL, CSFR3, DNMT3A, FLT3, GATA2, HRAS, IDH1, IDH2, JAK2, KIT, KRAS, MOL, MYD88, NPM1, NRAS, PTPN11, SETPB1, SF3B1, SRSF2, U2AF1, WT1, ASXL1, BCOR, CALR, CEBPA, ETV6, EZH2, IKZF1, NF1, PHF6, PRPF8, RB1, RUNX1, SH2B3, STAG2, TET2, TP53, ZRSR2, ALK, BCL2, BRAF, CCDN1, CREBBP, EGFR, ETV6, FGFR1, FGFR2, FUS, HMGA2, JAK2, KMT2A, MECOM, MET, MLLT10, MLLT3, MYBL1, MYH11, NTRK3, NUP214, PDGFRA, PDGFRB, RARA, RBM15, RUNX1, TCF3, TFE3*.

UK - *ANKRD26, ASXL1, BCOR, CALR, CBL, CEBPA, CSF3R, CUX1, DDX41, DNMT3A, ETV6, EZH2, FLT3, GATA1, GATA2, HRAS, IDH1, IDH2, IKZF1, JAK2, KIT, KMT2A, KRAS, MPL, NF1, NFE2, NPM1, NRAS, PHF6, PPM1D, PTPN11, RAD21, RUNX1, SETBP1, SF3B1, SH2B3, SRSF2, STAG2, TET2, TP53, U2AF1, WT1, ZRSR2*.

**Supplementary table 2. Second and further therapy lines in patients with non severe aplastic anemia.** HSCT hematopoietic stem cell transplant; ATG anti-thymocyte globulin; CyA cyclosporine; EPAG eltrombopag; MMF mycophenolate mofetil; TOX toxicity; CR complete response; PR partial response; NR no response; L lost at follow up.

| **First line** | **Gender** | **Age years** | **2nd line - response** | **3rd line - response** | **outcome** |
| --- | --- | --- | --- | --- | --- |
| **Cyclosporine** | F | 19 | MMF – NR | ATG - NR | ALIVE |
|  | M | 33 | HSCT - CR |  | L |
|  | M | 39 | EPAG - NR | HSCT - NR | DEAD |
|  | F | 48 | ATG – PR |  | ALIVE |
|  | F | 52 | ATG – PR |  | ALIVE |
|  | M | 58 | EPAG - PR |  | ALIVE |
|  | F | 59 | ATG - PR |  | ALIVE |
|  | M | 62 | ANDROGEN - TOX |  | L |
|  | M | 64 | ATG - NR | EPAG - NR | DEAD |
|  | F | 65 | ANDROGEN - PR |  | DEAD |
|  | F | 66 | ANDROGEN - NR | EPAG - NR | ALIVE |
|  | M | 67 | ANDROGEN - CR |  | ALIVE |
|  | M | 72 | EPAG - PR |  | ALIVE |
|  | M | 72 | EPAG - NR |  | DEAD |
|  | M | 73 | ATG - NR |  | L |
|  | F | 73 | ANDROGEN - PR |  | DEAD |
|  | F | 76 | EPAG - NR | TACROLIMUS - NR | ALIVE |
|  | M | 78 | EPAG - NR |  | DEAD |
| **ATG+Cyclosporine** | F | 16 | CYA - NR | HSCT - CR | ALIVE |
|  | F | 21 | TACROLIMUS - NR | ALEMTUZUMAB - PR | L |
|  | F | 21 | ATG - NR | CYA - CR | ALIVE |
|  | M | 22 | ATG - CR | TACROLIMUS - CR | L |
|  | M | 23 | ATG - CR | ANDROGEN - CR | L |
|  | M | 28 | HSCT - CR |  | DEAD |
|  | F | 31 | TACROLIMUS+EPAG - PR |  | ALIVE |
|  | F | 31 | HSCT - CR |  | ALIVE |
|  | M | 31 | HSCT - CR |  | ALIVE |
|  | M | 34 | TACR - CR |  | L |
|  | M | 37 | ATG+TACROLIMUS - NR | HSCT - CR | ALIVE |
|  | M | 42 | ATG+TACROLIMUS - NR | ANDROGEN - CR | L |
|  | F | 57 | EPAG - TOX | HSCT – CR | ALIVE |
|  | M | 58 | EPAG - NR |  | L |
|  | F | 64 | ATG + ANDROGEN - NR | ALEMTUZUMAB+EPAG - NR | L |
|  | M | 66 | ANDROGEN - NR | EPAG - NR | DEAD |
|  | M | 69 | ATG+TACROLIMUS - NR |  | L |
| **Cyclosporine+EPAG** | M | 69 | HSCT - NR |  | DEAD |
| **Alemtuzumab** | F | 67 | ANDROGEN- NR | EPAG - NR | ALIVE |
| **Tacrolimus** | M | 13 | ATG - CR |  | ALIVE |
| **Androgen** | M | 24 | ATG - NR | HSCT - CR | ALIVE |

**Supplementary table 3.** Multivariable analysis restricted to the last 10 years of observation (i.e. 2012 to 2022).

| **Variable** | **Hazard ratio** | **Standard error** | **Z** | **P** | **95% conf. interval** |
| --- | --- | --- | --- | --- | --- |
| Male gender | 2.804462 | 2.014335 | 1.44 | 0.151 | 0.68-11.46 |
| Age (decade) | 28.10422 | 34.68437 | 2.70 | 0.007 | 2.50-315.69 |
| Fibrosis MF-1 | 0.6721387 | 0.3269859 | -0.82 | 0.413 | 0.25-1.74 |
| PNH positivity | 0.0004606 | 0.0012382 | -2.86 | 0.004 | 2.37e-06-0.08 |
| Trilineage response | 2.90e-08 | 1.88e-07 | -2.67 | 0.007 | 8.69e-14-0.009 |
